# An exploration of the consequences of, and coping with loneliness in an ageing intellectual disability population

**DOI:** 10.12688/hrbopenres.13452.2

**Published:** 2022-07-05

**Authors:** Andrew Wormald, Philip McCallion, Mary McCarron

**Affiliations:** 1 School of Nursing and Midwifery, Trinity College Dublin, Dublin, Ireland; 2 College of Public Health, Temple University, Philadelphia, PA 19122, USA

**Keywords:** Loneliness, Intellectual Disability, Perceived Social Isolation, Learning Disability

## Abstract

**Background:** Loneliness has been associated with increased hypervigilance and sad passivity. The physiological and psychological reactions of people with an intellectual disability to loneliness have never been investigated. Therefore, this research aims to explore the outcomes of loneliness for an ageing intellectual disability population.

**Methods:** In Ireland, data from a nationally representative data set of people aged over 40 years with an intellectual disability (N=317) was applied to a social environment model that describes the effects of loneliness in five pre-disease pathways which are: health behaviours, exposure to stressful life events, coping, health and recuperation. The data was tested through chi-squared, ANCOVA and binary logistic regression.

**Results:** Being lonely predicted raised systolic blood pressure (A.O.R=2.051, p=0.039), sleeping difficulties (AOR=2.526, p=0.002) and confiding in staff (AOR=0.464 p=0.008). Additionally, participants who did 10 to 20 minutes of exercise daily (moderate activity) had significantly higher loneliness scores than those who did not (F=4.171, p<0.05).

**Conclusions: **The analysis supports the concept of hypervigilance in older people with an intellectual disability but finds that the health behaviours of the lonely do not differ from the not lonely. Future research needs to investigate the longitudinal relationships between loneliness and health

## Introduction

Loneliness is the distressing feeling that accompanies discrepancies between one's desired and actual social relationships ( Hawkley & Cacioppo, 2010). It is generally believed that the lonely tend not to seek help for their loneliness ( Perlman & Peplau, 1998), withdrawing from others and using coping strategies that perpetuate their situation ( Hawkley *et al.*, 2008) such as self-blaming ( Vanhalst *et al.*, 2015) and lack of trust ( Hensley *et al.*, 2012).

Loneliness in older people has negative consequences for health and wellbeing, being associated with increased metabolic dysregulation ( Shiovitz-Ezra & Parag, 2019) and increased systolic blood pressure (SBP) ( Hawkley *et al.*, 2010; Ong *et al.*, 2012). Moreover, loneliness has been reported as altering a person's lifestyle and physiological reactions to stress ( Hawkley & Cacioppo, 2007). In their social environment model of loneliness, Hawkley & Cacioppo (2007) argue that there are five pre-disease paths; health behaviours, exposure to stressful life events, coping style and support, physiology and recuperation, through which loneliness negatively influences a person's physiological resilience.

There is a growing body of evidence in older people to support each of the pre-disease pathways and their overall effect on physiological resilience. Hawkley & Cacioppo (2007) argue that unhealthy lifestyles contribute to poor health and early death in the first pathway health behaviours. Lonely people tend to be involved in more risky health behaviours ( Shankar, 2017) and have been found to exercise less, smoke ( Shankar *et al.*, 2011), take in more fats and calories, and are more likely to have a higher body mass index (BMI) ( Shiovitz-Ezra & Parag, 2019). It has been found that lonely people manage moods by eating, drinking and acting out sexually ( Hawkley & Cacioppo, 2007), and they attend general practitioners surgeries and accident and emergency departments more frequently than those who are not lonely ( Cleary, 2011). Contrary to this for people with an intellectual disability it has been reported that loneliness is not a predisposing variable for healthcare utilisation ( McCallion *et al.*, 2012).

In the second pathway, exposure to stressful life events, Hawkley & Cacioppo (2007) reported that the number of stressors experienced and the potency of those stressors are more prevalent in the lonely and diminish physiological resilience. Lonely people report being exposed to an increased number of stressful life events and ( Hawkley *et al.*, 2008) some argue that it is the accumulation of negative life events that lead to loneliness in older people ( Jylhä, 2004). However, the association between life events and loneliness is not consistently reported, and others have found no association ( Zebhauser *et al.*, 2015). The effect of each stressor is particular to an individual's circumstances. For instance, work stress has been reported to have more of an effect on unmarried people's loneliness ( Hawkley *et al.*, 2008). It is known that people with an intellectual disability experience more life events than found in the general population ( Gilmore & Cuskelly, 2014), it is not known if these relates to loneliness.

In the third path, coping style, lonely people are less likely to cope by seeking a confidant for support ( Victor *et al.,* 2008) and are more likely to regularly attend church ( Hawkley & Cacioppo, 2007). It is notable that people with an ID lack support from friends ( The Money, 2012), spouses or life partners. 

Consistent with the fourth pathway, the physiology of chronic stress in older people signal the vulnerability to disease. Chronic loneliness leads to activation of the autonomic nervous system, leading to heart rate and blood pressure increases ( Hawkley *et al.*, 2010). However, the effects of loneliness on cardiovascular health have recently been questioned, with one study claiming loneliness does not affect SBP ( Das, 2019). However, Das (2019) has been criticised for not considering the role of medications beyond baseline ( Hawkley & Schumm, 2019). Other researchers have reported that lonely people record a different cardiovascular response than non-lonely people in specific conditions ( Brown *et al.,* 2019). There is no evidence about physiological responses to loneliness in people with an ID.

Finally, recuperation counteracts the forces that drain physiological reserves. Lonely people have less effective sleep ( Coyle & Dugan, 2012), their sleep is more fragmented ( Kurina *et al.*, 2011) or altered ( Leigh-Hunt *et al.*, 2017), they take more time to go to sleep and have more night-time disturbances than non-lonely people ( Cacioppo *et al.*, 2000). Sleep difficulties and loneliness in people with an intellectual disability have both been found to be predictive of mental health difficulties ( Bond *et al.*, 2020) but no research has as yet studied the relationship between sleep and loneliness in this group.

Perlman & Peplau (1998) claimed people had four mechanisms for coping with loneliness; sad passivity, active solitude, spending money and social contact. The evidence available tends to support the concept of sad passivity being the most common coping mechanism. People with an ID tend to be atypical of the general population in their health behaviours. It has already been reported that loneliness is not a predictor of healthcare utilisation in this population ( McCallion *et al.,* 2012). In general, people with an ID have healthier diets, smoke less, and drink less alcohol than the general population, but they complete very little vigorous physical exercise, and 66% are classified as overweight or obese ( McCarron *et al.*, 2014). Consideration of loneliness in people with ID must consider these different patterns of coping. 

### This research

While there is a developed body of evidence that supports the effects of loneliness on physiological resilience in the ageing population, the cumulative findings do not come from a single data set, and there is very little evidence to suggest the findings apply to people with an intellectual disability. This research uses the five pathway social-environmental model to investigate the consequences of loneliness in terms of physical and psychological reactions and coping mechanisms. How the health-effects are experienced can be influenced by other variables such as gender ( Ward *et al.*, 2021) and functional limitations ( Wormald *et al.*, 2019) which will therefore be covariates in this study. Using one source, the Intellectual Disability Supplement to The Irish Longitudinal Study of Ageing (IDS-TILDA) dataset seeks to answer the questions: how do older people with an ID physically react to loneliness?; and do lonely people with an ID demonstrate the use of specific coping mechanisms?

## Methods

### Ethical considerations

Ethical approval was granted from the Faculty of Health Sciences research ethics committee in Trinity College Dublin and all services providers involved in the study. 

***Study design.*** The IDS-TILDA is a public patient involvement study that was codesigned with people with an intellectual disability and collects data from people aged over 40 years who are registered on the National Intellectual Disability Database (NIDD) about the ageing process. Data collection commenced in 2010. To date, three waves have been completed. The study encapsulated wide-ranging data including sociodemographic characteristics, social connectedness, physical and behavioural health, mental health, health care utilisation, employment and education, personal choices. The NIDD released 1800 personal identification numbers of potential participants, and the regional, national disability coordinator mailed invitation packs to each person. Participants were sent a consent pack, and where able, they self consented. Where people could not self consent, family/guardians consented on their behalf. Interviews are conducted directly with the participant, supported by a proxy or have the interview completed fully by a proxy. The proxy had to have known the participant for at least six months. Data was collected using a pre-interview questionnaire (PIQ), a face-to-face interview and a health fair. The PIQ was posted to the participants a week in advance of their face-to-face interview facilitating the participant to collect the required information and gain support for completion if required. The face-to-face interview utilised computer-assisted interviewing on encrypted laptops. The health fair was conducted separately from the main study and involved a researcher assessing eight health measures such as bone density, systolic blood pressure and weight.

This study is a cross-sectional analysis of wave 2 data. Wave 2 data was selected as it was the first wave to include the full 3-item i.e., a complete loneliness scale and use here prepares for future longitudinal comparisons to be addressed in future articles. In this study, we use the variables in the five pathway social-environmental model as dependent variables and test using the loneliness and social connectedness scale and co-variates as independent variables to understand the role of loneliness in each of these variables.

### Participants

Participants for the IDS-TILDA study had to be registered on the National Intellectual Disability Database and aged over 40 years at wave one in 2010. Wave 2 data collection was conducted in 2013 interviews in this study can be either by the participant alone or the participant may have a proxy supporting them. In this analysis, participants must have self-reported their answers to the loneliness questions and must have supplied their systolic blood pressure reading. Most other measures were usually self-reported.

### Measures

***Loneliness and social connectedness scale.*** The loneliness and social connectedness scale consisted of four items: The Three-Item Loneliness Scale ( Hughes *et al.,* 2004) and a self-labelling loneliness item. To aid in comprehension and to simplify the response options, the questions were divided into two parts. The first part had a lead-in of "Do you ever feel….." with a yes/no response. Only if participants responded yes to the first part did they receive the second part of the question asked, "how often do you feel….." with a three-point response set (rarely/sometimes/always). For each of the four items responses were coded 1 for responding no to the first question or for rarely/never to the second question, 2 for sometimes and 3 for almost always. Where a single item score was missing, data was imputed on a person-mean basis. A total of 35 people (11.0%) were missing a single item. The most commonly missed item was, "Do you ever feel isolated?" The scale demonstrated satisfactory internal consistency (Chronbach's alpha = .715) scores ranged from 4 to 12, and the mean score was 5.30 (SD = 1.58), a score of four meaning no feelings of loneliness and a score of 12 meant the person was lonely always across all four items.

For analysis in cross tabulations and the binary logistic regression the loneliness variable was dichotomised following the methodology of Pikhartova *et al.* (2016). Participants scoring in the bottom three quartiles scoring between 4 and 6 were categorised as not lonely (n=246), which equated to 77.6% of participants. Lonely participants were the top quartile who scored greater than six on the scale (n=71, 22.4%).

***Health variables.*** All health variables were taken from the second wave of data collection and were selected to approximate those described by Hawkley & Cacioppo (2007) in their social environment model (n=317 unless otherwise stated).

**Path 1 - Health behaviours**

Path 1 included four binary-coded variables, *Vigorous Activity, Moderate Activity, Mild Activity* and *Smoking*. Participant responses were coded one for yes and zero for no. A measure of *Body Mass Index* (BMI) (n=248, 78.2%) was created for each participant using either height and weight or ulna measurement ( Elia, 2003). Participants with a BMI of 30 or higher were classified as obese coded one. All others were coded zero. *Self-reported diet* (n=312, 98.45) was binary coded between excellent and very good, and good, fair or poor.

**Path 2 – Exposure to stressful events**

The life events scale used was an adapted version of the Hermans & Evenhuis (2012) life events scale for older people with intellectual disabilities. The scale here used 19 of the 28 items. Participants were asked if they had experienced any items on the list of life events in the previous 12 months. Participants who indicated the presence of a life event were then asked how stressful they found that life event. Stress was scored on a three-point scale; one, a lot, two, a little and three, none. In total, 311 (98.1%) participants responded to the scale. The numbers of *Life events* experienced over the previous 12 months were counted, and participants were classified as either high on the number of life events experienced or normal. Stresses were separated into three categories; *Social Stress, Relationship Stress* and *Service Stress*.

**Path 3 - Coping**

The coping mechanisms tested were being a *Church Attender* and *confiding* in different groups, family, friend, staff and other; responses were binary coded.

**Path 4 – Health**

*Systolic Blood Pressure* (SBP) (n=224, 70.6%) was measured using an Omron 10 device with the results binary coded into two categories; those with a score over 120mmHg were coded as high SBP, and those scoring below 120mmHg were classified as normal blood pressure.

**Path 5 – Recuperation**

There were four sleep variables *Trouble Falling Asleep (n=310, 97.8%), Interrupted Sleep (n=312, 98.4%), Waking Too Early (n=308, 97.1%)* and *Daytime Sleeping (n=310, 97.8%)*. Variables were dichotomised based on percentile. An overall *sleep scale* score was created by summing the scores of the four sleep items. Scores were binary coded between having difficulty sleeping and no difficulty sleeping.

***Co-variates.*** Functional limitations are measure using an 11 item self-reported scale aimed at measuring a participant's physical abilities. The scale was developed for use in the Health and Retirement Study ( Wallace *et al.*, 2004) and included questions such as "Please indicate the level of difficulty if any, you have with walking 100 yards" and "Please indicate the level of difficulty, if any, you have with bathing or showering." Participants were asked whether they had a problem doing each activity. Responses were scored one for no difficulty, two for some difficulty, three for a lot of difficulty, four for can't do it at all.

Gender was included as a co-variate

### Analysis

All statistical analysis was undertaken using SPSS v23.0.

Analysis followed the three-step approach undertaken by Lauder *et al.* (2006). Step 1, cross-tabulations were constructed. Each path variable was cross-tabulated, first with the loneliness scale variable and then the consistent loneliness variable. This produced proportions of the lonely that were relative to each variable. The data in the tables were tested for independence using chi-square.

Step 2, separate analyses of covariance (ANCOVA) were conducted. The ANCOVAs included the loneliness scale score as the dependent variable and a path variable as the independent variable. Functional limitations and gender were co-variates.

Step 3, binary logistic regression, was used to investigate loneliness's role as a predictor variable of each path variable. Functional limitations and gender were listed as co-variates. The Naglekerke R² statistic was calculated, excluding co-variates, for each path variable, where either the loneliness scale score or consistent loneliness was a significant predictor of a path variable. Calculating the Naglekerke R², in this manner, allows the fit of the loneliness variable to each health variable to be understood. Nagelkerke R² is one of the two pseudo-R² measures available in SPSS v 23.0 and offers the benefit over the Cox-Snell method of being scaled 0–1.

For all analysis, 95% bootstrap bias-corrected and accelerated confidence intervals were produced with 5,000 cases.

## Results

**Figure 1.**
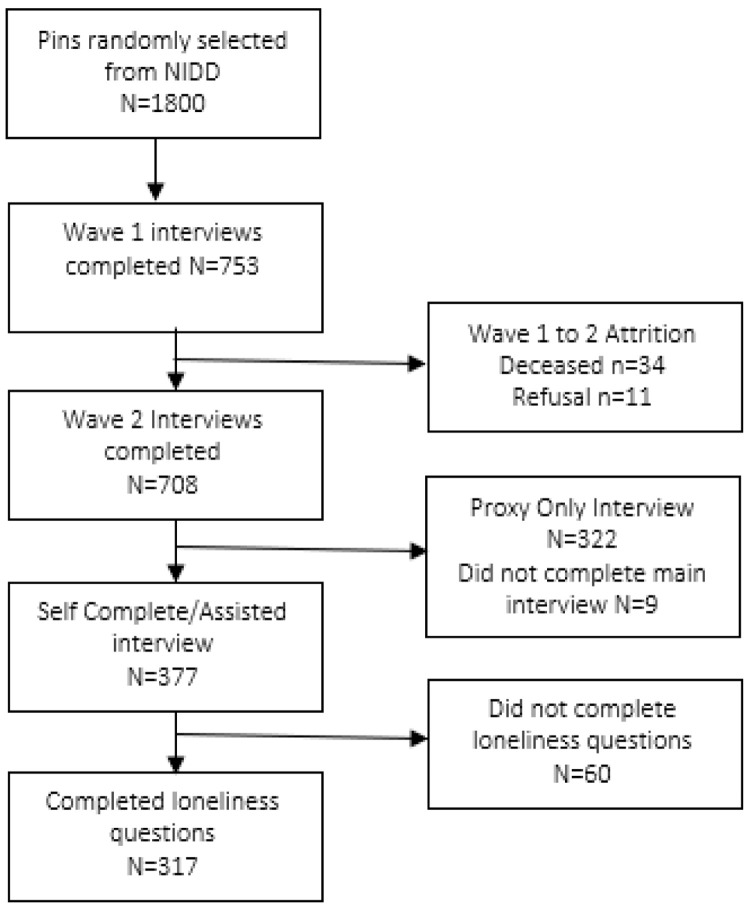
Flow diagram for participant inclusion in the loneliness research.

Participants for this study had to self-report their loneliness on the loneliness scale in wave two of data collection ( Figure 1). Table 1 represents the demographic breakdown of participants. Comparing those who completed the scale to those who did not complete the scale the average age of 56.16 (SD=8.578) was not significantly different for those who did not complete the scale (mean=56.95, SD=9.875). This subpopulation had a higher percentage of females (59.3%) than those that did not answer (46.3%), but there was no significant difference in the gender balance (χ²=2.691, p=0.101). There was an overrepresentation of those with mild and moderate disability in those who completed the loneliness scale compared to those who did not respond to the loneliness scale (χ²=179.190 p<0.001). 

**Table 1.** Demographic breakdown of participants answering the loneliness items in wave two (N=317). CI=confidence interval; ID=intellectual disability.

		Wave 2 Loneliness sample	%	Wave 2 others	%
Age (years)		56.16		56.95	
	95% CI Lower	55.22		55.96	
	95% CI Upper	57.11		57.94	
Sex					
	Male	129	40.7%	180	41.3%
	Female	188	59.3%	204	46.8%
	Unknown	0		52	11.9
Level of ID					
	Mild	119	37.5%	47	10.8%
	Moderate	154	48.6%	169	38.8%
	Other	11	3.4%	195	44.7%
	UnKnown	33	10.4%	25	5.7%

Cross-tabulations were calculated for each health variable against the dichotomised loneliness scale variable ( Table 2). All cells had an expected value of more than five participants, allowing chi-squared analysis to be conducted. Analysis of the loneliness scale score found in path 3, the not lonely on the loneliness scale were more likely to confide in staff (χ²=6.625 p<0.05). In path 4, those who were lonely were more likely to have raised SBP (χ²=4.424, p<0.05). Analysis of path 5 revealed that those who were lonely were proportionally more likely to have difficulties falling asleep (χ²=9.176, p<0.01) and waking too early (χ²=6.539, p<0.05).

**Table 2.** Cross-tabulations of independent variables with the loneliness scale.

Health Variable	Loneliness Scale				
		Yes	No	χ²	P
Path 1 Health Behaviour					
Vigorous Activity	Yes	6	23	0.054	0.817
	No	65	223		
Moderate Activity	Yes	23	59	2.032	0.134
	No	48	187		
Mild Activity	Yes	48	168	0.012	0.913
	No	23	78		
Smoke	Yes	9	22	0.881	0.348
	No	61	221		
Obese	Yes	31	96	0.299	0.585
	No	26	95		
Diet	Yes	42	152	0.182	0.669
	No	28	90		
Path 2 Stress Exposure					
Life Events	Yes	15	38	1.384	0.239
	No	54	204		
Relationship Changes	Yes	18	72	0.416	0.519
	No	53	174		
Social Changes	Yes	12	27	1.793	0.181
	No	59	219		
Service Stress	Yes	18	46	1.513	0.219
	No	53	200		
Path 3 Coping					
Attend Church	Yes	13	33	1.064	0.302
	No	58	213		
Confide in Family	Yes	23	73	0.193	0.660
	No	48	173		
Confide in Friend	Yes	12	25	2.427	0.119
	No	59	221		
Confide in Other	Yes	11	24	1.846	0.174
	No	60	222		
Confide in Staff	Yes	38	172	6.625	0.010
	No	33	74		
Path 4 Health Effects					
High SBP	Yes	32	75	4.424	0.035
	No	21	96		
Path 5 Recuperation					
Trouble Falling Asleep	Yes	21	35	9.176	0.002
	No	48	206		
Disturbed Sleep	Yes	31	79	3.223	0.073
	No	39	163		
Wake Too Early	Yes	19	35	6.539	0.011
	No	49	205		
Dozing	Yes	49	177	0.386	0.535
	No	21	63		
Sleep Scale	Yes	27	49	10.607	0.001
	No	41	191		

The mean loneliness scores were subject to ANCOVA, with gender and functional limitations being held constant ( Table 3). Those who had difficulty falling asleep tended to be lonelier (mean = 6.000) than those who did not have difficulty falling asleep (mean = 5.150). In path 1, participants who did moderate activity had significantly higher loneliness scores (mean = 5.524) than those who did not (mean = 5.221, F=4.171, p<0.05). There were no significant results in Path 2. In path 3, coping, those who confided in staff (mean = 5.152) had significantly lower scores on the loneliness scale than those who did not (mean = 5.589, F=5.716, p<0.05).

**Table 3.** Analysis of covariance of loneliness scores in each health variables.

Health Variable	Health Variable Response	Mean Loneliness Score	Sum of Squares	F	p
Path 1 Health Behaviour					
Vigorous Activity	Yes	5.310	0.142	0.055	0.815
	No	5.299			
Moderate Activity	Yes	5.524	10.670	4.171	0.042
	No	5.221			
Mild Activity	Yes	5.264	0.941	0.363	0.206
	No	5.376			
Smoke	Yes	5.687	9.244	3.604	0.059
	No	5.262			
Obese	Yes	5.433	2.703	0.964	0.327
	No	5.206			
Diet	Yes	5.284	0.050	0.019	0.890
	No	5.348			
Path 2 Stress Exposure					
Life Events	Yes	5.604	4.503	1.752	0.189
	No	5.236			
Relationship Changes	Yes	5.189	2.313	0.893	0.345
	No	5.343			
Social Changes	Yes	5.615	4.592	1.779	0.183
	No	5.255			
Service Stress	Yes	5.688	7.496	2.915	0.089
	No	5.201			
Path 3 Coping					
Attend Church	Yes	5.674	5.668	2.199	0.139
	No	5.236			
Confide in Family	Yes	5.313	0.095	0.037	0.848
	No	5.294			
Confide in Friend	Yes	5.730	4.431	1.716	0.191
	No	5.243			
Confide in Other	Yes	5.743	5.751	2.945	0.087
	No	5.245			
Confide in Staff	Yes	5.152	14.557	5.716	0.017
	No	5.589			
Path 4 Health Effects					
High SBP	Yes	5.570	10.207	3.551	0.061
	No	5.139			
Path 5 Recuperation					
Trouble Falling Asleep	Yes	6.000	34.455	13.907	<0.001
	No	5.150			
Disturbed Sleep	Yes	5.456	10.070	3.930	0.048
	No	5.178			
Wake Too Early	Yes	5.722	9.949	3.873	0.050
	No	5.213			
Dozing	Yes	5.266	1.150	0.442	0.507
	No	5.429			
Sleep Scale	Yes	5.816	25.841	10.284	0.001
	No	5.134			

Analysis of SBP in path 4 reveals that those who had high SBP scores had higher mean loneliness scores (mean=5.570) than those who were not lonely (mean=5.137), but differences were not significant (F=10.207, p>0.05). In path 5, those who had trouble falling asleep had significantly higher loneliness scores than those that did not have trouble falling asleep (F=13.907, p<0.01). People who reported having disturbed sleep (mean=5.456) had significantly higher loneliness scores than those who did not (mean=5.178, F=3.930, p<0.05). When the sleep scale individual items were combined, those who recorded higher scores had significantly higher scores on the loneliness scale (F=10.284, p<0.01).

The binary logistic regression results ( Table 4) indicate the strength of the loneliness variable's influence, with gender and functional limitations, held constant. The table details the adjusted odds ratio (AOR), the B statistic (the original scale coefficient), the standard error of the B value (created with bootstrap analysis), the bootstrap created confidence intervals, and the Naglekerk R², for variables where loneliness was a significant predictor.

**Table 4.** Binary Logistic regression for each path variable with the loneliness scale as a predictive variable.

		Bootstrap					
				95% Confidence Intervals			
Health Variable	AOR	B	S.E.	p	Lower	Upper	R²
Path 1 Health Behaviour							
Vigorous Activity	0.970	-0.030	0.958	0.946	-0.935	0.668	
Moderate Activity	1.873	0.627	0.333	0.054	-0.056	1.285	
Mild Activity	0.948	-0.053	0.312	0.867	-0.629	0.558	
Smoke	1.884	0.557	0.476	0.203	-0.382	1.397	
Obese	1.192	0.176	0.348	0.603	-0.513	0.870	
Diet	0.930	-0.072	0.295	0.798	-0.653	0.531	
Path 2 Stress Exposure							
Life Events	1.418	0.349	0.378	0.339	-0.433	1.043	
Relationship Stress	0.758	-0.277	0.345	0.403	-1.003	0.322	
Social Stress	1.627	0.487	0.428	0.231	-0.410	1.261	
Service Stress	1.359	0.307	0.344	0.353	-0.398	0.949	
Path 3 Coping							
Attend Church	1.319	0.277	0.375	0.450	-0.494	0.972	
Confide in Family	1.172	0.158	0.311	0.603	-0.487	0.765	
Confide in Friend	1.433	0.360	0.444	0.393	-0.549	1.104	
Confide in Other	1.673	0.515	0.413	0.181	-0.354	1.260	
Confide in Staff	0.464	-0.769	0.292	0.008	-1.333	-0.237	0.028
Path 4 Health Effects							
Systolic Blood Pressure	2.051	0.718	0.355	0.039	0.019	1.512	0.026
Path 5 Recuperation							
Falling Asleep	2.543	0.933	0.342	0.005	0.262	1.631	0.044
Disturbed Sleep	1.613	0.478	0.342	0.091	-0.100	1.073	
Wake Too Early	2.225	0.800	0.348	0.015	0.103	1.480	0.032
Dozing	0.858	-0.153	0.320	0.617	-0.764	0.534	
Sleep Scale	2.526	0.927	0.315	0.002	0.307	1.574	0.047

In path 3, those who were lonely were less than half as likely to confide in staff as the not lonely participants (AOR=0.464, B=-0.769, SE=0.292, 95% CI=-1.333, -0.237). Loneliness accounted for 2.8% of the confiding in staff variance (Naglekerke R²=.028).

Analysis of SBP in path 4 reveals that being categorised as lonely was a significant predictor of having raised SBP. The lonely were twice as likely to have raised blood pressure, with loneliness accounting for 2.6% of the SBP variance (AOR=2.051, B=0.718, SE=0.355, 95% CI =0.019, 1.512, Nagelkerke R²=0.026). In path 5, being lonely was a significant predictor of having trouble falling asleep (AOR=2.543, B=0.933, SE=0.342, 95% CI=0.262, 1.631). Loneliness accounted for 4.4% of the variance of the difficulty in falling asleep variable (Naglekerke R²=0.044). Loneliness was also a significant predictor of waking too early, accounting for 3.2% of the variance (AOR=2.225, B=0.800, SE=0.348, 95% CI =0.103, 1.480, Naglekerke R²=0.032). When the sleep scale individual items were combined, being lonely was a significant predictor of having sleep difficulties, accounting for 4.7% of the sleep scale variance (AOR=2.526, B=0.927, SE=0.315, 95% CI =0.307, 1.574, Nagelkerke R²=0.047).

## Discussion

This research offers the first evidence of how older people with an ID react to and cope with loneliness. The results indicate that older people with an ID reacted to loneliness with sleeping difficulties, raised systolic blood pressure, and were less likely to confide in staff/caregivers. The results also found that the lonely are more likely to take part in moderate physical activity.

This study supports previous research in the general population that indicated associations between loneliness and sleeping difficulties ( Kurina *et al.*, 2011) and loneliness and systolic blood pressure ( Hawkley & Cacioppo, 2007). These findings also extend previous knowledge ( Victor *et al.*, 2008), indicating the importance of whom people confide in over merely confiding as an act. Finally, for people ageing with an ID, the results disagree with the general ageing population's findings that lonely people are more likely to have worse health behaviours than non-lonely ( Hawkley & Cacioppo, 2007; Lauder *et al.*, 2006). There was no association found between health behaviours and loneliness, and only those who did moderate activity were found more likely to score higher on the loneliness scale.

The relationship found in this study between sleep and loneliness was consistent with research from the wider population. Loneliness often affects sleep because of the unconscious scanning for social threats caused by hypervigilance ( Hawkley & Cacioppo, 2010). When people have had their sleeping checked by the use of electronic devices such as nightcaps, researchers have found that lonelier participants have more disrupted sleep ( Kurina *et al.*, 2011), they take longer to fall asleep and have poorer sleep quality ( Cacioppo *et al.*, 2000). Sleep is an important precipitator of health problems as sleep counteracts the forces that drain the body, and lower quality sleep does not allow the restorative processes to operate. ( Hawkley & Cacioppo, 2007)

Hypervigilance is also reported to reduce sleep quality and be associated with increased vascular resistance ( Cacioppo & Cacioppo, 2014), which leads to increased SBP ( Hawkley & Cacioppo, 2007). Disrupted sleep has also been postulated as a causal route for raised SBP ( Bonnet & Arand, 2003).

Among people ageing with an ID, the lonely were twice as likely to have raised SBP. This evidence supports research from the wider population where it has been found that for every standard deviation rise in loneliness, SBP increased significantly ( Ong *et al.*, 2012). Although establishing a causal relationship remains for future research, a need is confirmed for attention to highlight blood pressure concerns among people with ID who report feelings of loneliness.

Hypervigilance has also been reported to cause people to be wary of others ( Cacioppo & Cacioppo, 2014). Here, whom a person confided in influenced their chances of becoming lonely and additionally, being lonely influenced whom people confided in, creating a cycle of protection or harm. More specifically, those who confided in staff were less likely to be lonely, and those who were lonely were more than one and a half times more likely to confide in others. It is possible that people who confided in staff were confiding in someone who could make a difference to underlying issues, whereas others may not affect a person's circumstances directly.

In this study we found that those who took part in moderate activity scored significantly higher on the loneliness scale. Emotional and instrumental support has been found to enable engagement in physical exercise ( Rackow *et al.*, 2015). Considering this general population finding, one possible explanation for the moderate activity finding here is that compared to the wider population many of the people with intellectual disabilities in our study receive support from care workers ( McCausland *et al.*, 2018). Staff may have encouraged those they suspected of being lonely to engage in more exercise. The role of staff and other caregivers and linkage between increased physical activity and loneliness needs further investigation.

## Implications

The findings here present a complex interaction between health issues and loneliness. Too often the presence of health symptoms results in assessment of health issues alone when assessment that includes loneliness and other psychosocial concerns may offer a better perspective on what needs to be addressed. Rather than professionals seen occasionally and focused on health concerns, it is care staff who are more likely to pick up concerns sleeping difficulties that may more likely be related to isolation and loneliness. Consistency by providers in assigning care staff will increase the likelihood of observing and reporting concerns and person centered planning to improve the quality of life and may be the most helpful approaches.

## Limitations

In analysing the data as reactions to and coping with loneliness, this research may imply causality. To show causality, three criteria need to be met; covariation, temporal ordering and elimination of competing theories ( Hayes, 2013). This analysis cannot prove causality since it is not an experiment controlling the above conditions; however, steps have been taken in the analysis to approximate the three criteria. Covariation was dealt with through the type of analysis conducted that showed the variables did have covariation. Two competing theories were accounted for through the utilisation of the co-variates gender and functional limitations. There are more than two possibilities for competing causes of the variables health paths, and these need to be considered with further investigation of the data. This analysis is the first work to look at how older people with an intellectual disability react to and cope with loneliness, and some results confirmed findings from the general population, further suggesting validity. Additional investigation assuming findings hold will add to the validity of the findings.

Likert type scales have been reported as problematic for people with an intellectual disability ( Gilmore & Cuskelly, 2014). However, others have found that people with an ID are capable of reliably answering three-point scales ( Stancliffe *et al.*, 2014), and the UCLA loneliness scale ( Russell, 1996) has been found to adequately represent loneliness in those with cerebral palsy ( Balandin *et al.*, 2006).

The data used here were taken at a single time point and, therefore, do not have the support of longitudinal analysis. Unfortunately, the full loneliness scale was only available from wave two of data in the IDS-TILDA study. Further analysis will offer a more detailed insight as further waves of data also incorporating the scale become available. 

The data collection techniques employed in this study meant that only people who could self-report their feelings are represented. This limitation excludes any understanding of loneliness in those who have difficulty communicating. In future research, alternative research methodologies must be employed to help those with communication difficulties express their feelings of loneliness.

## Conclusion

This study was the first to explore reactions to and coping with loneliness in an ageing population of people with an ID. This study used a social environment model of loneliness described by Hawkley & Cacioppo (2007) that analysed the effects of loneliness on physiological resilience through five pre-disease paths. The results add support to path 4 (health effects) and path 5 (recuperation). They extend parts of path 3 (coping) but find little supporting evidence for paths 1 (health behaviours) and path 2 (exposure to stress). The analysis undertaken supports the concept of hypervigilance and suggests that it is experienced in this population, leading to sleep disruption, raised SBP and wariness of other people. The analysis does not support any hypothesised coping mechanisms ( Perlman & Peplau, 1998), finding no differences in the health behaviours between the lonely and the non-lonely. Future research needs to investigate the longitudinal relationships of loneliness and health in this ageing ID population.

## Data availability

### Underlying data

The data controller for this project is Trinity College Dublin

Approval for data sharing was not sought at ethics approval stage nor was it included in the study information and consent forms provided to participants. The anonymised underlying data for this paper is available in a restricted format. Access to data which could potentially pose a risk to the confidentiality of IDS-TILDA participants has been withheld following assessment of sample size, cell counts and the data context.

Anonymised data and study documentation may be accessed through the Irish Social Science Data Archive (ISSDA) at https://www.ucd.ie/issda/data/ids-tilda/. To access the data, please complete a ISSDA Data Request Form for Research Purposes, sign it, and send it to ISSDA by email.

For teaching purposes, please complete the ISSDA Data Request Form for Teaching Purposes, and follow the procedures, as above. Teaching requests are approved on a once-off module/workshop basis. Subsequent occurrences of the module/workshop require a new teaching request form.

Data will be disseminated on receipt of a fully completed, signed form. Incomplete or unsigned forms will be returned to the data requester for completion.

### Extended data

Trinity’s Access to Research Archive: IDS-TILDA Wave 2: Main Interview Questionnaire, https://doi.org/10.25546/96788.

Trinity’s Access to Research Archive: IDS-TILDA Wave 2: Pre-Interview Questionnaire, https://doi.org/10.25546/96789.

Data are available under the terms of the Creative Commons Attribution 4.0 International license (CC-BY 4.0).
