# Supplementary material for: An exploration of the consequences of, and coping with loneliness in an ageing intellectual disability population
Source: HRB Open Res. 2022 Jul 5;5:2. Originally published 2022 Jan 11. [Version 2] doi: 10.12688/hrbopenres.13452.2 (PMC9315231; doi:10.12688/hrbopenres.13452.2)
Supplement: Peer review history

# Peer review: Reviewer response for version 2

**DOI:** 10.21956/hrbopenres.14844.r32451

**Published:** 2022-07-25

**Authors:** Owen Doody

**Affiliations:** 1 Department of Nursing and Midwifery, North Bank Campus, University of Limerick (UL), Limerick, Ireland

All comments and revisions have been taken on board and a clear, well present papers is evident.

Is the work clearly and accurately presented and does it cite the current literature?

Yes

If applicable, is the statistical analysis and its interpretation appropriate?

Yes

Are all the source data underlying the results available to ensure full reproducibility?

Yes

Is the study design appropriate and is the work technically sound?

Yes

Are the conclusions drawn adequately supported by the results?

Yes

Are sufficient details of methods and analysis provided to allow replication by others?

Yes

Reviewer Expertise:

Intellectual disability, community support, nursing care.

I confirm that I have read this submission and believe that I have an appropriate level of expertise to confirm that it is of an acceptable scientific standard.

# Peer review: Reviewer response for version 2

**DOI:** 10.21956/hrbopenres.14844.r32450

**Published:** 2022-07-21

**Authors:** Ewelina Rydzewska

**Affiliations:** 1 University of Edinburgh, Edinburgh, UK

I would like to thank the authors for addressing my comments so diligently and thoroughly. I'm satisfied with the amendments.

Is the work clearly and accurately presented and does it cite the current literature?

Partly

If applicable, is the statistical analysis and its interpretation appropriate?

Yes

Are all the source data underlying the results available to ensure full reproducibility?

Yes

Is the study design appropriate and is the work technically sound?

Yes

Are the conclusions drawn adequately supported by the results?

Partly

Are sufficient details of methods and analysis provided to allow replication by others?

Partly

Reviewer Expertise:

Public health, health of people with ID

I confirm that I have read this submission and believe that I have an appropriate level of expertise to confirm that it is of an acceptable scientific standard.

# Peer review: Reviewer response for version 1

**DOI:** 10.21956/hrbopenres.14660.r31569

**Published:** 2022-03-21

**Authors:** Ewelina Rydzewska

**Affiliations:** 1 University of Edinburgh, Edinburgh, UK

**Abstract:**

1. '(...) in five pre-disease pathways...' - missing punctuation makes this sentence unclear.
2. In the last sentence of the results, can you clarify who was the comparison group? The phrase 'moderate activity' already suggests a relatively high level of activity, but maybe this is just my impression of the wording.
3. In the first sentence of the conclusions, do you mean that there aren't enough ID-specific support initiatives? Please, clarify your point if possible.

**Introduction:**

1. The first paragraph is not really needed, as you make the same points in the next few paragraphs.
2. You seem to refer to ID-specific studies availability or lack thereof only when discussing the third and fourth pathway. Can you please comment on other pathways too?
3. 'The evidence available tends to support the concept of sad passivity being the most common coping mechanism.' - Can you please explain further how you reached this conclusion?

**Methods:**

1. Please, explain the acronyms 'NIDD' and 'PINS'.
2. Can you please add information on the data controller for IDS-TILDA?
3. I think that the comments on Likert type scales etc. belong more in your limitations section, not in your methods.
4. *Vigorous Activity, Moderate Activity, Mild Activity* and *Smoking - *what were the binary responses for these variables? Yes or no?
5. 'Participants who had experienced a life event were asked how stressful they found the event and were scored as to the level of stress experienced.' - this sentence repeats what you already state earlier in the paragraph.
6. An overall *sleep scale* score was created. - can you say a bit more about this?
7. Information on literature pertaining to relevant covariates belongs more in the introduction, not in your methods. Here you should only state what covariates you've chosen for the analysis.

**Results:**

1. 'Table 1 represents the demographic breakdown of participants, comparing those who completed the scale to those who did not complete the scale.' - I can only see the stats for those who completed the scale?

**Discussion:**

1. I would advise exercising caution about making assumptions about health behaviours in this population, as you did not look at behaviours such as eating habits, drinking alcohol or taking drugs if I understood correctly? Can the fact that those who did moderate exercise had higher loneliness scores than those who did not can be partially explained by different residential patterns and/or access to additional support in the ID population? People with ID often live in supported living contexts, as opposed to the general population, and may have access to additional types of support from care workers etc. who may encourage them to engage in more exercise/activity where the person with ID is perceived to be isolated from peers. Isolated people in the general population often won't have that kind of support/external motivation to exercise.

**Limitations:**

1. You mention that participants must have self-reported their answers to the loneliness questions to be included in the study. Was any other information proxy-reported for the included cases?

Is the work clearly and accurately presented and does it cite the current literature?

Partly

If applicable, is the statistical analysis and its interpretation appropriate?

Yes

Are all the source data underlying the results available to ensure full reproducibility?

Yes

Is the study design appropriate and is the work technically sound?

Yes

Are the conclusions drawn adequately supported by the results?

Partly

Are sufficient details of methods and analysis provided to allow replication by others?

Partly

Reviewer Expertise:

Public health, health of people with ID

I confirm that I have read this submission and believe that I have an appropriate level of expertise to confirm that it is of an acceptable scientific standard, however I have significant reservations, as outlined above.

# Response

**Published:** 2022-06-29

**Authors:** Andrew Wormald

**Affiliations:** Trinity College Dublin, Ireland

Dear Reviewer

 Thank you for taking the time to review this article. We have taken note of your constructive and helpful comments and we have acted upon each of them. You will find below a list of the amendments that have been included in the article based on your comments. Where we mention page numbers in our comments we are referring to the page number that can be found at the bottom of the page e.g. page 1 as they appear in the current document.

 We hope you find these changes now bring the paper up to the required standard.

 Many Thanks

 The Authors

**Reviewer 2**

- Abstract:

'(...) in five pre-disease pathways...' - missing punctuation makes this sentence unclear.

 Thankyou, we have modified the sentence to read (page 2), **"in five pre-disease pathways which are:"**

- In the last sentence of the results, can you clarify who was the comparison group? The phrase 'moderate activity' already suggests a relatively high level of activity, but maybe this is just my impression of the wording.

To clarify this sentence, we have amended the wording around moderate exercise to read (Page 2): **Additionally, participants who did 10 to 20 minutes of exercise daily (moderate activity) had significantly higher loneliness scores than those who did not (F=4.171, p<0.05).**

- In the first sentence of the conclusions, do you mean that there aren't enough ID-specific support initiatives? Please, clarify your point if possible.

We have modified the first sentence of the conclusion on page 2 to read: **The analysis supports there is a need for loneliness-focused hypervigilance among older people with an intellectual disability but supports that the health behaviours of the lonely do not differ from the not lonely.**

- Introduction: The first paragraph is not really needed, as you make the same points in the next few paragraphs.

** We have deleted the first paragraph as per your recommendation**

- You seem to refer to ID-specific studies availability or lack thereof only when discussing the third and fourth pathway. Can you please comment on other pathways too?

Path 1 added (page 3) **: Contrary to this for people with an intellectual disability it has been reported that loneliness is not a predisposing variable for healthcare utilisation**

 Path 2 (Page 3): **It is known that people with an intellectual disability experience more life events than found in the general population** **(Gilmore & Cuskelly, 2014)** **, it is not known if these relates to loneliness.**

 Path 5 (page 3): **Sleep difficulties and loneliness in people with an intellectual disability have both been found to be predictive of mental health difficulties** **(Bond et al., 2020)** **but no research has as yet studied the relationship between sleep and loneliness in this group.**

- 'The evidence available tends to support the concept of sad passivity being the most common coping mechanism.' - Can you please explain further how you reached this conclusion?

Thank you for pointing out your understanding of this statement. In the general population loneliness has been found to influence health behaviours in a way that fits the description of sad passivity. However, in this study there is no evidence to support the claim that loneliness has any influence on health behaviours of people with an intellectual disability. We have modified the paragraph on page 16 to read:

**The findings here present a complex interaction between health issues and loneliness. Too often the presence of health symptoms results in assessment of health issues alone when assessment that includes loneliness and other psychosocial concerns may offer a better perspective on what needs to be addressed. Rather than professionals seen occasionally and focused on health concerns, it is care staff who are more likely to pick up concerns sleeping difficulties that may more likely be related to isolation and loneliness. Consistency by providers in assigning care staff will increase the likelihood of observing and reporting concerns and person centred planning to improve the quality of life and may be the most helpful approaches.**

- Methods: Please, explain the acronyms 'NIDD' and 'PINS'.

Thankyou for highlighting these unexplained acronyms NIDD is the National Intellectual Disability Database, we have added this to the text on page 4. PINS is personal identification numbers, we have changed the text from PINS.

- Can you please add information on the data controller for IDS-TILDA?

In the *Underlying data* section on page 18 we have added: **The data controller for this project is Trinity College** **Dublin**

- I think that the comments on Likert type scales etc. belong more in your limitations section, not in your methods.

Thank you for this insight. We have moved this paragraph to the limitations section on page 17.

- Vigorous Activity, Moderate Activity, Mild Activity and Smoking - what were the binary responses for these variables? Yes or no?

To clear up this issue we have added the following sentence in Path 1 – Health behaviours on page 6: **Participant responses were coded one for yes and zero for no.**

- 'Participants who had experienced a life event were asked how stressful they found the event and were scored as to the level of stress experienced.' - this sentence repeats what you already state earlier in the paragraph.

 Thank you for highlighting this repetition we have deleted the sentence.

- An overall sleep scale score was created. - can you say a bit more about this?

We have modified the sleep sentence in Path 5 Recuperation on page 6 to give a more detailed explanation: **An overall *sleep scale* score was created by summing the scores of the four sleep items**

- Information on literature pertaining to relevant covariates belongs more in the introduction, not in your methods. Here you should only state what covariates you've chosen for the analysis.

Thank you for highlighting this omission. We have removed the supporting information for the covariates and have added the following sentence on page 4: **How the health-effects are experienced can be influenced by other variables such as gender** **(Ward et al., 2021)** **and functional limitations** **(Wormald et al., 2019)** **which will therefore be covariates in this study**

 Results:

- 'Table 1 represents the demographic breakdown of participants, comparing those who completed the scale to those who did not complete the scale.' - I can only see the stats for those who completed the scale?

Thank you for highlighting this grammatical issue we have modified table 1 to include the results of those who did not take part and the sentences in the Results section on page 7 to read: **Table 1** **represents the demographic breakdown of participants. Comparing those who completed the scale to those who did not complete the scale the average age of 56.16 (SD=8.578) was not significantly different for those who did not complete the scale (mean=56.95, SD=9.875).**

- Discussion: I would advise exercising caution about making assumptions about health behaviours in this population, as you did not look at behaviours such as eating habits, drinking alcohol or taking drugs if I understood correctly? Can the fact that those who did moderate exercise had higher loneliness scores than those who did not can be partially explained by different residential patterns and/or access to additional support in the ID population? People with ID often live in supported living contexts, as opposed to the general population, and may have access to additional types of support from care workers etc. who may encourage them to engage in more exercise/activity where the person with ID is perceived to be isolated from peers. Isolated people in the general population often won't have that kind of support/external motivation to exercise.

Thank you, you make very valid points. The discussion does say on page 16 “ *There is no evidence in this study that loneliness influenced the health behaviours of people with an intellectual disability*” We have also added the wording on page 16:

**In this study we found that those who took part in moderate activity scored significantly higher on the loneliness scale. Emotional and instrumental support has been found to enable engagement in physical exercise** **(Rackow *et al*., 2015)** **. Considering this general population finding, one possible explanation for the moderate activity finding here is that compared to the wider population many of the people with intellectual disabilities in our study receive support from care workers** **(McCausland et al., 2018)** **. Staff may have encouraged those they suspected of being lonely to engage in more exercise. The role of staff and other caregivers and linkage between increased physical activity and loneliness needs further investigation**

- Limitations: You mention that participants must have self-reported their answers to the loneliness questions to be included in the study. Was any other information proxy-reported for the included cases?

To help clarify this question we have added the following details to the participants section on page 5. **Wave 2 data collection was conducted in 2013 interviews in this study can be either by the participant alone or the participant may have a proxy supporting them. In this analysis, participants must have self-reported their answers to the loneliness questions and must have supplied their systolic blood pressure reading. Most other measures were usually self-reported.**

# Peer review: Reviewer response for version 1

**DOI:** 10.21956/hrbopenres.14660.r31326

**Published:** 2022-02-11

**Authors:** Owen Doody

**Affiliations:** 1 Department of Nursing and Midwifery, North Bank Campus, University of Limerick (UL), Limerick, Ireland

**Abstract**

- Minor flow/readability aspects to be addressed. 

**Introduction** 

- Repeating needs to be omitted e.g. increased systolic blood pressure paragraphs 1 and 3.
- While accepting little research pertaining to intellectual disability (ID) and loneliness it would be good to get a handle as to what population you have to draw your information from is it older persons, mental health, etc.

**Methods** 

- Give justification for using wave 2 data which was 2013 and SPSS 23 which was 2015, why are we analyzing data from 2013 which is 9 years old especially when there is a wave 3, and if using SPSS 23 was the analysis done a number of years ago?
- Measures - give 'n =' and '%' but not for all, please give both.

**Findings** 

- Participants who did moderate activity had significantly higher loneliness scores (higher than all other groups of activity levels - is it saying those who did moderate activity more likely to be lonely over those who do not or, low activity? And if so, bring out in discussion).

**Discussion** 

- Bring out implications is little research done based on your results what needs to happen e.g. if blood pressure or sleep issues present should a loneliness assessment be conducted or what strategies should be looked at to support people with ID and bring this out for the all the results you discuss i.e. implications.

Is the work clearly and accurately presented and does it cite the current literature?

Yes

If applicable, is the statistical analysis and its interpretation appropriate?

Yes

Are all the source data underlying the results available to ensure full reproducibility?

Yes

Is the study design appropriate and is the work technically sound?

Yes

Are the conclusions drawn adequately supported by the results?

Yes

Are sufficient details of methods and analysis provided to allow replication by others?

Yes

Reviewer Expertise:

Intellectual disability, community support, nursing care.

I confirm that I have read this submission and believe that I have an appropriate level of expertise to confirm that it is of an acceptable scientific standard, however I have significant reservations, as outlined above.

# Response

**Published:** 2022-06-29

**Authors:** Andrew Wormald

**Affiliations:** Trinity College Dublin, Ireland

Dear Reviewer

 Thank you for taking the time to review this article. We have taken note of your constructive and helpful comments and we have acted upon each of them. You will find below a list of the amendments that have been included in the article based on your comments. Where we mention page numbers in our comments we are referring to the page number that can be found at the bottom of the page e.g. page 1 as they appear in the current document.

 We hope you find these changes now bring the paper up to the required standard.

 Many Thanks

 The Authors

**Reviewer 1**

- Abstract. Minor flow/readability aspects to be addressed.

Thank you, we have amended the abstract to improve readability.

- Introduction. Repeating needs to be omitted e.g. increased systolic blood pressure paragraphs 1 and 3.

We removed paragraph 1 from the introduction (page 2) which has removed the repeating of items in the literature.

- While accepting little research pertaining to intellectual disability (ID) and loneliness it would be good to get a handle as to what population you have to draw your information from is it older persons, mental health, etc.

The information in the literature review is generally drawn from the wider ageing population We have ensured that throughout the literature review we have specified the characteristics of the populations reported upon (pages 2 to 4). For example, on page 3 we say **“There is a growing body of evidence in older people to support each of the pre-disease pathways and their overall effect on physiological resilience. Hawkley & Cacioppo (2007)”.**

- Methods. Give justification for using wave 2 data which was 2013 and SPSS 23 which was 2015, why are we analyzing data from 2013 which is 9 years old especially when there is a wave 3, and if using SPSS 23 was the analysis done a number of years ago?

Wave 2 data was used so that a baseline for future longitudinal analysis could be established. Wave 2 was the first wave to include the complete loneliness scale. Analysis of this data began with SPSS 23 to meet grant reporting and thesis requirements but was not prepared for an article until more recently.

 We have added the following text to the Study Design section on page 5: **Wave 2 data was selected as it was the first wave to include the full 3-item i.e., a complete loneliness scale and use here prepares for future longitudinal comparisons to be addressed in future articles. **

- Measures - give 'n =' and '%' but not for all, please give both. 

Within the Measures section we have ensured that each variable has the n and the % for example we have modified the information on Body mass index on page 6 to read: **A measure of Body Mass Index (BMI) (n=248, 78.2%) was created for each participant using either height and weight or ulna measurement (Elia, 2003).**

- Findings. Participants who did moderate activity had significantly higher loneliness scores (higher than all other groups of activity levels - is it saying those who did moderate activity more likely to be lonely over those who do not or, low activity? And if so, bring out in discussion).

Thank you for highlighting this issue - we have now included the following in the discussion (page 16) about physical activity.

**In this study we found that those who took part in moderate activity scored significantly higher on the loneliness scale. Emotional and instrumental support has been found to enable engagement in physical exercise** **(Rackow *et al*., 2015)** **. Considering this general population finding, one possible explanation for the moderate activity finding here is that compared to the wider population many of the people with intellectual disabilities in our study receive support from care workers** **(McCausland et al., 2018)** **. Staff may have encouraged those they suspected of being lonely to engage in more exercise. The role of staff and other caregivers and linkage between increased physical activity and loneliness needs further investigation**

- Discussion. Bring out implications is little research done based on your results what needs to happen e.g. if blood pressure or sleep issues present should a loneliness assessment be conducted or what strategies should be looked at to support people with ID and bring this out for the all the results you discuss i.e. implications.

On page 17 we have added the following implications to the discussion:

**The findings here present a complex interaction between health issues and loneliness. Too often the presence of health symptoms results in assessment of health issues alone when assessment that includes loneliness and other psychosocial concerns may offer a better perspective on what needs to be addressed. Rather than professionals seen occasionally and focused on health concerns, it is care staff who are more likely to pick up concerns about sleeping difficulties that may more likely be related to isolation and loneliness. Consistency by providers in assigning care staff will increase the likelihood of observing and reporting concerns and person centred planning to improve the quality of life and may be the most helpful approaches.**
